# Exogenous 6-Benzyladenine Improved the Ear Differentiation of Waterlogged Summer Maize by Regulating the Metabolism of Hormone and Sugar

**DOI:** 10.3389/fpls.2022.848989

**Published:** 2022-04-07

**Authors:** Juan Hu, Baizhao Ren, Yuhai Chen, Peng Liu, Bin Zhao, Jiwang Zhang

**Affiliations:** State Key Laboratory of Crop Biology and College of Agronomy, Shandong Agricultural University, Tai’an, China

**Keywords:** summer maize, waterlogging, ear differentiation, yield, sucrose-cleaving enzymes

## Abstract

Waterlogging (W-B) is a major abiotic stress during the growth cycle of maize production in Huang-huai-hai plain of China, threatening food security. A wide range of studies suggests that the application of 6-benzyladenine (6-BA) can mitigate the W-B effects on crops. However, the mechanisms underlying this process remain unclear. In this study, the application of 6-BA that effectively increased the yield of summer maize was confirmed to be related to the hormone and sugar metabolism. At the florets differentiation stage, application of 6-BA increased the content of trans-zeatin (TZ, + 59.3%) and salicylic acid (SA, + 285.5%) of ears to induce the activity of invertase, thus establishing sink strength. During the phase of sexual organ formation, the TZ content of ear leaves, spike nodes, and ears was increased by 24.2, 64.2, and 46.1%, respectively, in W-B treatment, compared with that of W. Accordingly, the sugar metabolism of summer maize was also improved. Therefore, the structure of the spike node was improved, promoting the translocation of carbon assimilations toward the ears and the development of ears and filaments. Thus the number of fertilized florets, grain number, and yield were increased by the application of 6-BA.

## Introduction

Given the ongoing increases in global temperature, the frequency and intensity of extreme climate weather have increased, which cause marked damage to crops and food system infrastructure, with the potential to destabilize food systems and threaten local to global food security, especially the developing countries (Lesk et al., 2016). Changes in temporal and spatial patterns of precipitation directly influence the crop water cycle, and consequently, drought and waterlogging (W-B) stresses are increasingly becoming important factors that constrain crop yield (Tao et al., 2003a,b). Globally, around 12% of the world’s arable lands were affected by extreme rainfall events-induced W-B leading to approximately 20% of crop yield reduction (Setter and Waters, 2003). A continuous increase in population coupled with the increasing frequency and intensity of extreme rainfall events results in serious food security problems. Therefore, there is an urgent need for mitigation strategies to reduce the reduction of yield induced by W-B.

Maize is most vulnerable to climate variability and changes among the staple crops as the maize yields are significantly correlated with the seasonal precipitation in the North China Plain (Tao et al., 2008). Therefore, developing effective strategies to cope with the climatic risk in maize production is of great significance to ensuring food security. Previous studies suggested that W-B impedes maize roots’ growth and decreases the photosynthetic capability of the shoot and plant productivity. However, plant productivity is also determined by carbohydrate allocation, other than photosynthetic capacity (Moore et al., 2021). The ear is the main harvesting organ of maize, thus the growth and development of ears largely reflect the plant productivity of maize. The formation of maize ears is a dynamic development process of sequential differentiation. The maize ear is composed of spikelet pairs arranged in rings sequentially initiated at the ear apex. The ear growth cone can continuously differentiate into spikelet pairs until the sexual organ formation period (Bonnett, 1940; Kiesselbach, 1949). However, under adversity conditions, the insufficient supply of nutrients may reduce the differentiation time and the differentiation rate of spikelets, decreasing the total number of florets. Therefore, the ability of carbohydrate translocation to ears is a key for plant productivity.

Carbohydrate translocation from photosynthetic source tissues to non-photosynthetic sink tissues is in the form of sucrose. The entry of carbon from sucrose into cellular metabolism such as the synthesis of starch, protein, and cellulose in plants can potentially be catalyzed by either sucrose synthase or invertase. Sucrose translocation and sink strength are mediated by the sucrose-cleaving enzymes (sucrose invertase and sucrose synthase). The high activity of sucrose invertase is pivotal to ensure sufficient reducing sugar for spike differentiation and development in summer maize, thus ensuring sufficient florets to develop into grains (Hu et al., 2021a). It has been confirmed that increasing the sucrose invertase is an effective strategy to improve the source and sink relationship, thus increasing the crop yield in the context of adverse conditions (Julius et al., 2017; Xu et al., 2020).

Many investigations have recently been conducted to provide insights into sugar signaling and its interplay with hormones in the fine-tuning of plant growth, development, and survival (Sakr et al., 2018). Abscisic acid (ABA) regulates many plant adaptive responses to environmental constraints (Rohde et al., 2000; Finkelstein et al., 2002; Vishwakarma et al., 2017). Additionally, many mutations affecting sugar sensing and signaling are allelic to genes encoding components of the ABA synthesis or ABA transduction pathways suggesting that ABA and sugars can cross-influence each other (Laby et al., 2000; Rook et al., 2001; Brocard-Gifford et al., 2004; Akihiro et al., 2005). Moderate drought can increase the ABA content of stems, thus promoting the remobilization of carbohydrates into ears, increasing the grain-filling rate (Abid et al., 2017). GAs can mediate the photosynthetic (Tuna et al., 2008; Jiang et al., 2012) and sugar metabolic activities (Miyamoto et al., 1993, 2000; Chen et al., 1994; Mehouachi et al., 1996; Ranwala and Miller, 2008; Choubane et al., 2012; Liu et al., 2015; Machado et al., 2017), thus directly regulating the plant carbon status. Additionally, cytokinins and sugars acted both agonistically and antagonistically on gene expression, cross-influencing their metabolism and transport (Barbier et al., 2015; Wang et al., 2016), which may impinge on signaling pathways. Glucose has been confirmed to strongly affect genes involved in cytokinin metabolism and signaling (Kushwah and Laxmi, 2014). Besides, extensive crosstalk between sugars and other hormones such as brassinosteroids, ethylene, strigolactones, and auxin also function in regulating plant growth and development (Sakr et al., 2018).

The plant growth regulators are widely used in plant adaption to abiotic stresses in recent years. They can modulate the content and balance of endogenous hormones, regulate the synthesis and translocation of carbohydrates, and improve the source and sink relationship, thus playing a wide range of roles in plant growth and development. For example, the application of cytokinins can increase the florets of maize by counteracting the inhibition of ABA on sucrose translocation; application of SA can promote the translocation of photosynthesis assimilates toward ears, thus increasing the grain yield (Thompson, 2014; Kang et al., 2021). Our previous studies suggested that the application of 6-benzyladenine (6-BA) increased the yield of waterlogged summer maize from many aspects such as improving photosynthesis, balancing the metabolism of carbon and nitrogen, and delaying the leaf senescence (Ren et al., 2017). In addition, the 6-BA effects on endogenous hormones have been widely discussed. Moreover, the hormonal regulation of the source-sink relationship has been studied (Albacete et al., 2014). However, less is known regarding the effects of 6-BA on carbon translocation and spike differentiation of waterlogged summer maize that are directly related to the grain yield. Therefore, in this study, we focused on the effects of 6-BA on the ear differentiation processes, the changes of endogenous hormones, the carbon partitioning between source and sink tissues, and the cross-talk between hormones and sugars to find out the underlying mechanism of 6-BA, improving the grain yield of waterlogged summer maize.

## Materials and Methods

### Experimental Site and Conditions

Field experiments were conducted at an experimental farm of Shandong Agricultural University (36.09°N, 117.09°E) in the 2017 and 2018 cropping seasons. The effective cumulative temperatures (temperature > 10^°^C was defined as effective temperature) of summer maize growth periods during 2017 and 2018 were 1,857.5 and 1,836.2^°^C day, respectively. On average, 479.6 mm of precipitation occupied 72.8% of the total annual precipitation that occurred during the summer maize growth periods. N, P, and K fertilizers were applied as base fertilizer: 210 kg ha^–1^ N (urea, 46% N), 75 kg ha^–1^ P_2_O_5_ (calcium superphosphate, 17% P_2_O_5_), and 150 kg ha^–1^ K_2_O (muriate of potash, 60% K_2_O). Disease, weeds, and pests were well controlled in each treatment according to the management of the local farmers.

The experiment was designed as a randomized block experiment using the summer maize hybrid DengHai 605 (DH605). Four treatments, namely, waterlogging (W), spraying 6-BA after W-B, no waterlogging stress (CK), and spraying 6-BA on non-waterlogged plants (CK-B) were set in this study. The W-B stress was imposed at the third leaf stage, and the duration of W-B treatment was 6 days. In total, 100 mg L^–1^ 6-BA was applied as foliar sprays at the rate of 150 ± 5 ml per plant on all leaves, from 16:00 to 18:00 the next day after W-B, whereas the remaining plots were sprayed with deionized water. Each treatment was replicated three times. The plot size was 4 m × 4 m. Each plot was separated by polyvinyl chloride boards partitions. The water used to maintain the W-B condition was applied through water pipes. The water level was maintained at 2–3 cm above the soil surface in waterlogged treatments. Diseases and pests were well monitored and controlled.

### Ear Differentiation Characteristics

Maize was sown on 15 June 2017 and 8 June 2018 with a plant density of 67,500 plants ha^–1^. Five representative plants were sampled from each plot at the jointing growth stage (5th–6th leaf stage, V5–V6), booting stages (12th–13th leaf stage, V12–V13), and sexual organ formation stage (13th leaf stage to tasseling stage, V13–VT) to determine the stages of tassel and ear development. The husks around the growth cone were stripped with a dissecting needle, and then the development processes of ears were observed and photographed with a stereoscopic microscope.

On the fifth day after fertilization, five ears from each plot were sampled to measure ear floret differentiation. The fertilized florets, degeneration florets, and well-developed florets were distinguished and recorded according to the method described by Cui et al. (2015).

### The Structure of Filaments

Before the fertilization of the filaments, filaments of apical florets from three plants of each plot were sampled and preserved in stationary liquid for structural analysis using a scanning electron microscope.

### The Structure of Spike Node

Three spike nodes were sampled from each plot and preserved in the formaldehyde-acetic acid-ethanol fixative (40% formalin: glacial acetic acid: 70% ethanol, 1:1:18, by vol). The samples were dehydrated in a graded alcohol series. The materials were cut using a thin blade and stained with safranine. The slices were observed and photographed using a biological microscope (NI-u; Nikon, Tokyo, Japan). The number and area of bundles were measured. A total of 30 slices were observed and measured per plot.

### ^13^C Pulse Labeling, Sampling, and Analysis

At the 12th leaf stage, five plants from each plot were labeled according to the method described by Hu et al. (2021b) using ^13^CO_2_. At the VT stage, three ^13^CO_2_ labeled plants were sampled and separated into stems, leaves, ear leaves, spike nodes, shanks, ears, and tassels. These samples were dried, weighed, and ball-milled for analysis (Schussler and Westgate, 1994).

### Assays for the Activities of Superoxide Dismutase, Peroxidase, Catalase, and the Content of Malonaldehyde

Before the fertilization of the filaments, filaments from three plants of each plot were sampled. The activities of superoxide dismutase (SOD), peroxidase (POD), and catalase (CAT) and the content of malonaldehyde (MDA) were measured according to the method described by Hu et al. (2020).

### Extraction and Quantification of Abscisic Acid, Trans-Zeatin, Salicylic Acid, and Jasmonic Acid

At the florets differentiation processes, eight ears from each plot were sampled; during the phase of sexual organ formation, ear leaves, spike nodes, spike handles, and ears from three plants of each plot were sampled. These samples were frozen in liquid nitrogen and stored at –80^°^C for the quantification of hormones. The contents of hormones were extracted and quantified using a triple quadrupole mass spectrometer (ACQUITYUPLCI-Class/XevoTQ-S, Waters, Milford, MA, United States) equipped with an electrospray ion source according to the method described by Engelberth et al. (2003).

### Activity Assays for Invertase and Sucrose Synthase

Before the phase of sexual organ formation, eight ears from each plot were sampled; during the phase of sexual organ formation, ear leaves, spike nodes, shanks, and ears from three plants of each plot were sampled. The activities of sucrose invertase and sucrose synthase were measured according to the method described by Hu et al. (2021b).

### Assay of Soluble Sugar, Sucrose, and Starch Contents

During the phase of sexual organ formation, three plants were sampled from each plot and separated into stems, leaves, ear leaves, spike nodes, shanks, ears, and tassels. The ear leaves, spike nodes, shanks, and ears were dried, weighed, and ball-milled for the determination of sugar contents. Sugars were extracted by deionized water through the boiling water bath, and the starch was extracted using 2N HClO_4_ by boiling water bath according to the method described by Hanft and Jones (1986).

### Yield and Yield Components

A total of 30 ears from the middle three rows of each plot were harvested to measure yield and yield components (moisture content was approximately 14%).

### Data Analysis

IBM SPSS Statistics 21.0 (IBM Corporation, Armonk, NY, United States) was used for data statistics and analysis. Sigma plot 10.0 (Systat Software, Inc., Richmond, CA, United States) was used for data processing and plotting. Comparisons among groups were tested by one-way ANOVA and LSD tests, and differences between the means were considered significant at *P* < 0.05.

## Results

### The Effects of 6-Benzyladenine on the Grain Yield of Waterlogged Summer Maize

W-B significantly impaired the plant growth, resulting in a decrease in grain yield. However, the application of 6-BA could improve the plant growth and grain yield of waterlogged summer maize (Supplementary Figure 1). W-B decreased the kernels per ear and 1,000-kernels weight by 20.6 and 5.1%, respectively, thus leading to 24.1% grain yield losses. The application of 6-BA effectively increased the grain yield of waterlogged summer maize. The kernels per ear and grain yield of CK-B were 7.5 and 13.3%, respectively, higher than those of CK; the kernels per ear, 1,000-kernels weight, and grain yield of W-B were 18.6, 5.4, and 25.0%, respectively, higher than those of W (Table 1).

**TABLE 1:** The effects of 6-BA on the yield of waterlogged summer maize.

Year	Treatments	Kernels per ear	1,000-kernels weight	Grain yield
2017	CK	553ab	363a	13,193b
	CK-B	582a	370a	14,768a
	W	425c	349b	9,270d
	W-B	522b	368a	12,192c
2018	CK	540b	364a	13,270b
	CK-B	593a	367a	15,205a
	W	443d	341b	9,985d
	W-B	507c	359a	11,872c

*Different letters on bars indicate the significant differences among treatments at P < 0.05 using the LSD test.*

### The Effects of 6-Benzyladenine on the Ear Differentiation Characteristic of Waterlogged Summer Maize

#### The Ear Differentiation Processes

W-B significantly decreased the ear differentiation processes; the initiation of spike differentiation was delayed by 4 days after W-B. In addition, the period of spike differentiation to initiation of filament elongation was shortened around 1 day. Application of 6-BA had no significant effects on the ear differentiation process. Moreover, W-B impeded the development of the apical florets and decreased the elongation rate of filaments. However, the application of 6-BA promoted apical florets development and filament elongation of waterlogged summer maize (Supplementary Figures 2, 3).

#### The Total Florets and Fertilized Florets

On average, there were 493, 524, 399, and 459 florets differentiated before the initiation of filament elongation in CK, CK-B, W, and W-B, respectively. Accordingly, W-B decreased the total florets by 15.2%, compared with that of CK. Application of 6-BA increased the total florets by 2.5% in CK-B, compared with that of CK. In addition, the total florets of W-B were 12.0% higher than that of W. Besides, the fertilized florets were decreased by 21.0% in W, compared with that of CK. Application of 6-BA increased the fertilized florets by 21.6%, compared with that of W (Table 2).

**TABLE 2:** The effects of 6-BA on the differentiation and fertilization of florets.

Year	Treatment	The florets differentiated at T1 stage	The florets differentiated at T2 stage	Fertilized florets
2017	CK	568b	848a	627b
	CK-B	632a	909a	672a
	W	392c	736b	534d
	W-B	544d	832a	615c
2018	CK	497b	909a	723b
	CK-B	525a	924a	776a
	W	406d	759c	533d
	W-B	455c	841b	682c

*Different letters on bars indicate the significant differences among treatments at P < 0.05 using the LSD test.*

#### The Activities of Superoxide Dismutase, Peroxidase, and Catalase; The Content of Malonaldehyde; And the Structure of Filaments

**FIGURE 1:**
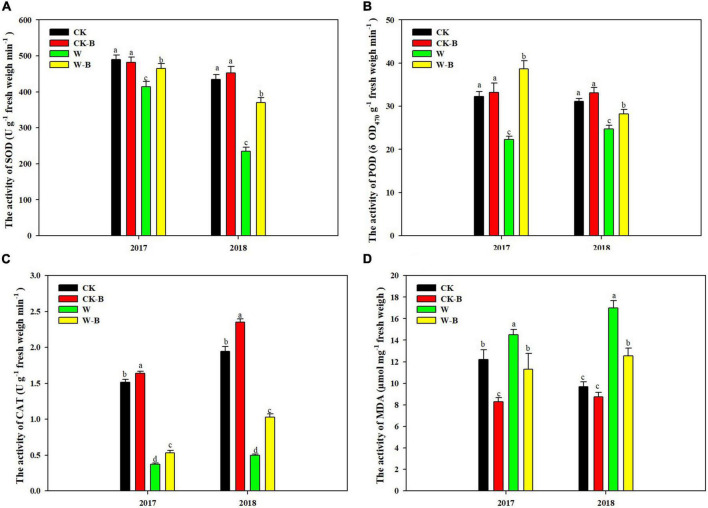
The effects of 6-BA on the activities of antioxidant enzymes and the content of MDA in the filaments of apical florets. **(A)** SOD; **(B)** POD; **(C)** CAT; **(D)** MDA. CK, control, no waterlogging stress; CK-B, spraying 6-BA on non-waterlogged plants; W, waterlogging; W-B, spraying 6-BA after waterlogging. Different letters on bars indicate the significant differences among treatments at *P* < 0.05 using the LSD test.

The activities of SOD, POD, and CAT of filaments were reduced by 30.7, 25.8, and 74.8%, respectively, in waterlogged summer maize, compared with those of CK. However, their activities were 35.0, 44.0, and 74.1% higher in W-B, respectively, than those of W. Accordingly, the MDA content was increased by 47.3% in W, compared with that of CK, while decreased by 24.2% in W-B, compared with that of W. In addition, W-B damaged the structure of filaments (Figure 1). In W treatment, the surface of filaments was horizontally wrinkled, the floral hairs fall on the filament surface, and the structure of stigma was wrinkled. However, the application of 6-BA mitigated the adverse effects of W-B on the filament structure (Supplementary Figure 4).

### The Effects of 6-Benzyladenine on the Carbohydrate Partition and Spike Node Structure

#### The ^13^C Translocation

**FIGURE 2:**
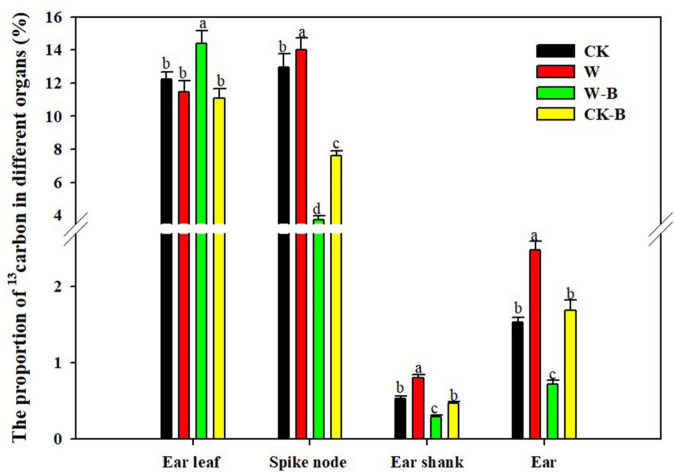
The effects of 6-BA on the ^13^C distribution in summer maize. CK, control, no waterlogging stress; CK-B, spraying 6-BA on non-waterlogged plants; W, waterlogging; W-B, spraying 6-BA after waterlogging. Different letters on bars indicate the significant differences among treatments at *P* < 0.05 using the LSD test.

W-B significantly increased the proportion of ^13^C in ear leaves by 17.7%, compared with that of CK, but decreased the proportion of ^13^C in spike node, shank, and ear by 71.5, 46.5, and 53.1%, respectively. However, the application of 6-BA increased the proportion of ^13^C in spike node, shank, and ear by 7.9, 50.4, and 62.3%, respectively, in CK-B, compared with that of CK. The proportion of ^13^C in spike node, shank, and ear of W-B was increased by 105.2, 64.0, and 135.9%, respectively, in W-B, compared with that of W (Figure 2).

#### The Structure of Spike Node

W-B significantly decreased the large and small bundles of spike nodes by 18.1 and 18.9%, respectively, compared with that of CK. Application of 6-BA increased the large and small bundles by 21.8 and 7.4%, respectively, in CK-B, compared with that of CK. Similarly, the large and small bundles of W-B were increased by 17.1 and 17.2%, respectively, compared with that of W (Table 3 and Supplementary Figure 5).

**TABLE 3:** The effects of 6-BA on the structure of spike nodes.

Year	Treatment	The number of small bundles	The number of large bundles	The area of the large bundles	The area of sieve tubes
2017	CK	243a	118b	0.171b	0.019b
	CK-B	263a	149a	0.185a	0.021a
	W	198c	98c	0.116d	0.012d
	W-B	226b	115b	0.160c	0.014c
2018	CK	245a	129b	0.199a	0.020a
	CK-B	262a	151a	0.197a	0.021a
	W	198c	103d	0.126c	0.013c
	W-B	239b	121c	0.169b	0.016b

*Different letters on bars indicate the significant differences among treatments at P < 0.05 using the LSD test.*

### The Effects of 6-Benzyladenine on Hormones of Summer Maize Plant

**FIGURE 3:**
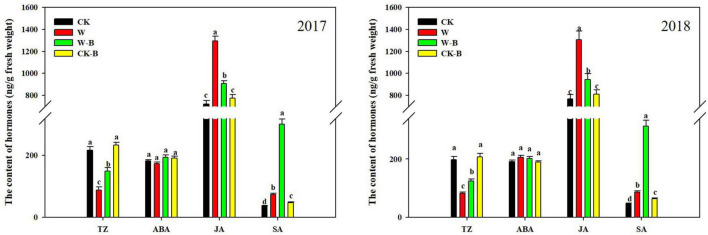
The effects of 6-BA on the hormones metabolism of ears at the T1 stage. CK, control, no waterlogging stress; CK-B, spraying 6-BA on non-waterlogged plants; W, waterlogging; W-B, spraying 6-BA after waterlogging. Different letters on bars indicate the significant differences among treatments at *P* < 0.05 using the LSD test.

At the T1 stage, the content of trans-Zeatin (TZ) in waterlogged summer maize was 58.8% lower; however, the contents of jasmonic acid (JA) and salicylic acid (SA) were 75.0 and 96.1%, respectively, higher than those of CK. The ratio of ABA/TZ, JA/TZ, and SA/TZ was significantly increased by 145.2, 324.3, and 375.6%, respectively, in W treatment, compared with that of CK. Application of 6-BA mitigated the decrease of TZ in ears of waterlogged summer maize. Additionally, the JA content of W-B was 28.8% lower than that of W, but the content of SA was 285.5% higher than that of W. The ratio of ABA/TZ and JA/TZ was significantly decreased by the application of 6-BA (Figure 3).

**FIGURE 4:**
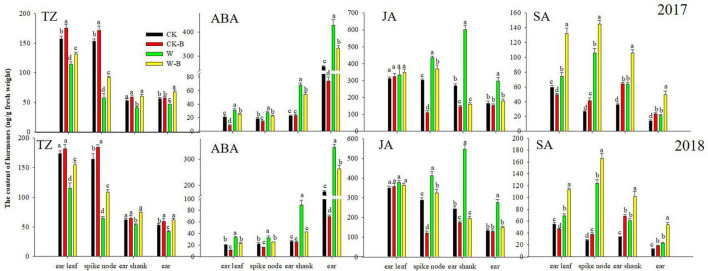
The contents of hormones in different organs at the T2 stage. CK, control, no waterlogging stress; CK-B, spraying 6-BA on non-waterlogged plants; W, waterlogging; W-B, spraying 6-BA after waterlogging. Different letters on bars indicate the significant differences among treatments at *P* < 0.05 using the LSD test.

At the T2 stage, the content of TZ (–30.3%) was decreased; however, the contents of ABA (+57.8%) and SA (+25.4%) were increased in ear leaves of W treatment, compared with those of CK. Additionally, the contents of TZ in W treatment were decreased by 61.6 and 17.7%, respectively, in spike node and shank. However, the contents of ABA, JA, and SA were increased. While the content of TZ was decreased by 19.0% in the ear, the contents of ABA, JA, and SA were increased by 81.4, 94.2, and 71.5%, respectively. Application of 6-BA increased the contents of TZ and SA but decreased the ABA and JA contents of spike node, shanks, and ear in W-B, compared with those of W. The ratio of ABA/TZ and JA/TZ was effectively decreased, but the ratio of SA/TZ of W-B was increased, compared with that of W (Figure 4).

### The Effects of 6-Benzyladenine on Sucrose-Cleaving Enzymes Activities

**FIGURE 5:**
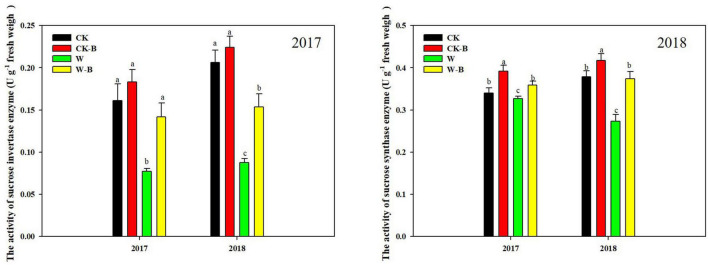
The effects of 6-BA on the activities of sucrose-cleaving enzymes at T1 stage. CK, control, no waterlogging stress; CK-B, spraying 6-BA on non-waterlogged plants; W, waterlogging; W-B, spraying 6-BA after waterlogging. Different letters on bars indicate the significant differences among treatments at *P* < 0.05 using the LSD test.

**FIGURE 6:**
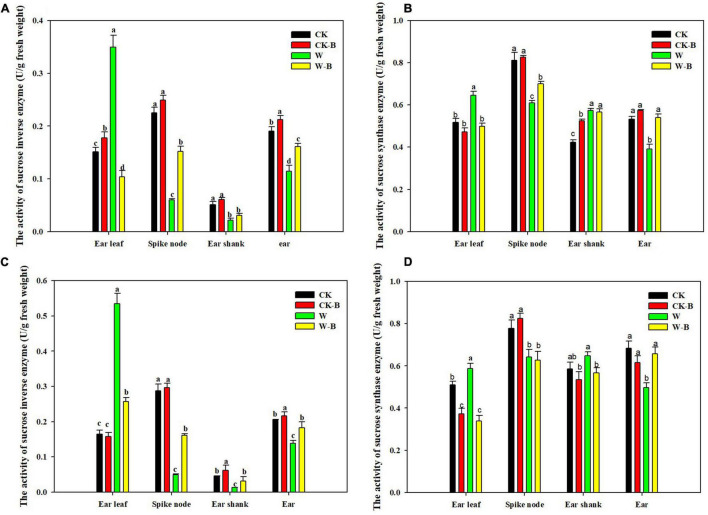
The effects of 6-BA on the activities of sucrose-cleaving enzymes at T2 stage. **(A)** (2017), **(C)** (2018), The activities of sucrose invertase enzyme in 2017 and 2018; **(B)** (2017), **(D)** (2018), The activities of sucrose synthase enzyme in 2017 and 2018. CK, control, no waterlogging stress; CK-B, spraying 6-BA on non-waterlogged plants; W, waterlogging; W-B, spraying 6-BA after waterlogging. Different letters on bars indicate the significant differences among treatments at *P* < 0.05 using the LSD test.

At the T1 stage, the activity of sucrose invertase and sucrose synthase of ears was decreased by 54.7 and 15.9%, respectively, in waterlogged summer maize, compared with that of CK. However, the application of 6-BA increased their activities of ears by 79.2 and 23.4%, respectively, in W-B, compared with that of W (Figure 5). At the T2 stage, W-B increased the activities of sucrose invertase and synthase enzymes in ear leaves but decreased their activities in spike node and ear. Application of 6-BA effectively mitigated the increases in sucrose invertase (–61.1%) and synthase activity (–32.5%) in ear leaves and the decreases in waterlogged summer maize in spike node and ear (Figure 6).

### The Effects of 6-Benzyladenine on the Sugar Contents and Starch Content of Summer Maize at the T2 Stage

**FIGURE 7:**
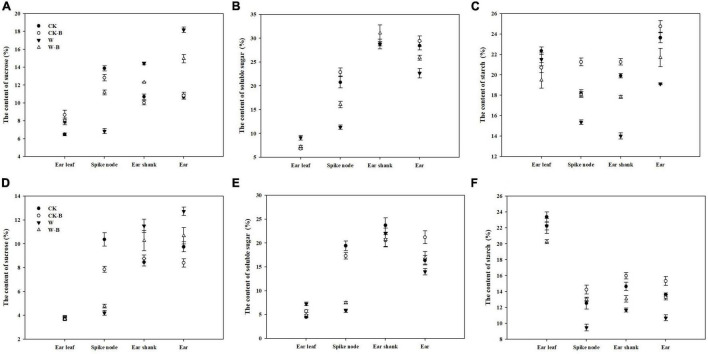
The effects of 6-BA on the contents of sugars and starch in 2017 and 2018. **(A)** (2017), **(C)** (2018), The contents of sucrose in 2017 and 2018; **(B)** (2017), **(D)** (2018), The contents of soluble sugar in 2017 and 2018. **(E)** (2017), **(F)** (2018), The contents of starch in 2017 and 2018. CK, control, no waterlogging stress; CK-B, spraying 6-BA on non-waterlogged plants; W, waterlogging; W-B, spraying 6-BA after waterlogging.

The content of sucrose in ear leaves was not significantly affected by W-B, while the content of soluble sugar was increased. In addition, W-B significantly increased the sucrose contents of the ear shank (+35.5%) and ear (+50.3%) but decreased their soluble sugar contents (–3.7 and –17.4%, respectively), compared with those of CK. The contents of starch in the spike node, ear shank, and ear were also significantly decreased by W-B. Application of 6-BA effectively decreased the soluble sugar contents of ear leaves (–20.0%) and increased the soluble sugar contents of the spike node and ear by 78.3 and 17.6%, respectively. Additionally, the starch contents of spike node, ear shank, and ear in W-B were increased by 27.3, 19.7, and 18.9%, respectively, compared with those of W (Figure 7).

### The Correlation Analysis

**FIGURE 8:**
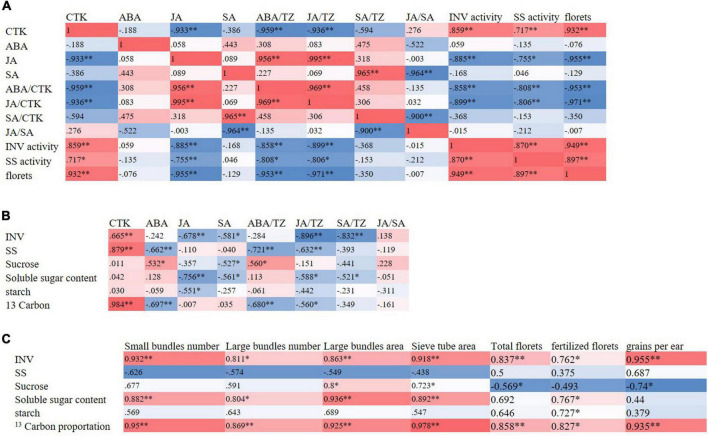
Correlation heat maps. According to the correlation coefficient value of hormones contents, sucrose-cleaving enzymes, sucrose contents, ^13^C distributions, and yields and yield components, the thermal map (heat map) was drawn. Red (1) represents a significant positive correlation and blue (-1) represents a significant negative correlation. The darker the color of the color block, the stronger the correlation. **(A)** Correlation heat map of hormone contents with sucrose cleaving enzymes and ear traits in ears at the T1 stage, **(B)** correlation heat map of hormone contents with sugar metabolism and ^13^C distribution characteristics in spike nodes and ears at the T2 stage, **(C)** correlation heat map of sugar metabolism and ^13^C distribution characteristics with spike node structure and ear characteristics at T2 stage. INV, sucrose invertase; SS, sucrose synthase enzyme; T1, florets differentiation stage; T2, sexual organ formation stage; * and ** respectively indicate the significant differences among treatments at *P* < 0.05 and *P* < 0.01 using the LSD test.

At the T1 stage, the activities of sucrose-cleaving enzymes were positively correlated with the content of cytokinin in ears and negatively correlated with the content of JA, the ratio of ABA/TZ, and JA/TZ. Significantly, the number of florets was positively correlated with the activities of sucrose-cleaving enzymes in the ears. At the T2 stage, the number of total and fertilized ear florets and the kernels per ear were positively correlated with the number and area of bundles in spike nodes. The number and area of bundles in spike nodes were positively correlated with the proportion of ^13^C in spike nodes. In addition, the proportion of ^13^C in the spike node was positively correlated with the activity of sucrose invertase in the spike node. Moreover, the activity of sucrose invertase in spike nodes was positively correlated with the content of cytokinin in spike nodes and negatively correlated with the ratio of JA/TZ and SA/TZ (Figure 8).

## Discussion

Grain yield of maize was mainly determined by the kernels per ear and 1,000-kernel weight. Significantly, the kernels per ear were more vulnerable to climate variability and change (Gao et al., 1989). The decrease of kernels per ear contributed to about 70% of W-B-induced grain yield losses (Hu et al., 2021a). The kernels were developed from florets. Therefore, the florets’ differentiation processes were the key to the formation of final mature kernels per ear. The paired spikelets and florets differentiated before the T2 stage can grow well and were likely to develop into mature kernels. However, after entering the sexual organ formation period, especially in the meiotic period, the differentiation of spikelets and florets tends to be unbalanced and more florets tend to develop into degradation or deformed florets (Zheng et al., 1990). In this study, W-B significantly decreased the number of florets at the T1 stage. Although W-B had less effect on the number of florets that formed during the period of sexual organ formation, the development of apical florets and the growth of filaments were significantly affected by W-B, leading to an increased pollination time gap between the basal and apical kernels. As a result, the ability of florets differentiated at the T2 stage to develop into kernels was significantly decreased. Therefore, promoting the florets differentiation during the T1 stage and balancing the differentiation of spikelets and florets at the T2 stage were crucial to determining the final mature kernels per ear. Interestingly, we found that the application of 6-BA could effectively increase the number of florets at the T1 stage, promote the growth and development of apical spikelets and florets, and improve the growth and structure of filaments, thus increasing the locus of effective florets and grain yield of waterlogged summer maize.

The ear differentiation processes are extensively regulated by hormones. Studies have proved that the content of cytokinins in ears of summer maize was peaked at the spikelet differentiation stage suggesting the pivotal role of cytokinin in florets differentiation. In addition, the high ratio of JA/CTK, ABA/CTK contributed to the abortion of florets (Wang, 2019). Besides, SA has been reported to act as a positive role in florets differentiation. In this study, W-B significantly decreased the content of cytokinin and SA in the ears, but increased the content of JA, which contributed to the impediment of florets differentiation. Previous studies have confirmed that the application of 6-BA could effectively mitigate the W-B effects on hormones (Hu et al., 2021b). In consistent with our previous study, Gao et al. (2007) reported that the application of 6-BA could modulate the endogenous cytokinin and ABA contents by promoting and inhibiting their biosynthesis. Additionally, cytokinin treatment has also been reported to elevate SA production by directly inducing the expression of isochorismate synthase (Wildermuth et al., 2001; Gaille et al., 2002, 2003; Choi et al., 2010). Moreover, the interplay between hormones is extensively established (Takei et al., 2004; Tsai et al., 2012), suggesting that the application of 6-BA could affect the metabolism of endogenous hormones in a wide range. Furthermore, the application of 6-BA could regulate MAPK and phosphorus signals to do a long effect on the synthesis and signal transduction pathways of hormones (Hu et al., 2021b). In consistent with these studies, in this study, application of 6-BA effectively mitigated the decrease or increase of cytokinin, SA, and JA in ears at the T1 stage, which further proved that application of 6-BA could promote the differentiation of florets contributing to the improvement of grain growth by doing a long effect on the metabolism of hormones.

Moreover, since florets differentiation was associated with active growth or the activation of biological processes, a possible link to the carbohydrate supply had been suggested (Kuiper, 1993). Carbohydrates were translocated from source (leaf) toward sink (ear) in the form of sucrose (Evert, 1982). The ability of an organ or a tissue to unload carbohydrates from the phloem defined its sink strength. Enzymes involved in the immediate sucrose metabolism are expected to be important both for phloem unloading and for the import of sucrose into sink organs (Ho et al., 1991). The sucrose must be cleaved into glucose and fructose to function in sink metabolism pathways. In this study, the number of florets at the T1 stage was positively correlated with the sucrose-cleaving enzymes of the ears. However, the activities of sucrose-cleaving enzymes in ears at the T1 stage were substantially lower in waterlogged summer maize, thus impeding the entry of carbon from sucrose into cellular metabolism. The cross-talk of sugar metabolism and hormones had been widely discussed. Cytokinin and SA have been confirmed to induce invertase activities, thus particularly participating in regulating sink strength, photosynthate partitioning, and phloem unloading (Ehness et al., 1997; LeClere et al., 2008). However, the high content of ABA and JA could inhibit the activities of sucrose invertase and transporters, thus impeding the long-distance transport of carbohydrates (Parish et al., 2012). In this study, the activity of sucrose invertase in the ears was positively correlated with the content of cytokinin but negatively correlated with the ratio of JA/CTK and ABA/CTK. These results underlie a close connection between hormones, the activities of sucrose-cleaving enzymes, and the floret differentiation processes. To some extent, it confirmed that the application of 6-BA improved the metabolism of endogenous hormones to regulate the activities of sucrose-cleaving enzymes, thus promoting the differentiation of florets at the T1 stage.

For increasing the effective florets differentiated during the T2 stage, it was critical to improve the plant nutrition status. The blocked nutrient supply to ears during this period affected the development of florets, especially the apical florets as the supply of assimilates to the apical kernels was inferior to the supply to the middle and basal kernels (Shen et al., 2018). Furthermore, the fertilization of the basal, oldest ovaries can stop the development of younger, apical ovaries and cause their abortion (Freier et al., 1984; Cárcova and Otegui, 2001). In this study, W-B significantly decreased the content of cytokinin and increased the content of JA and SA, thus decreasing the activities of sucrose invertase and synthase, leading to the lower proportion of ^13^C in spike nodes. Accordingly, the contents of sucrose, soluble sugar, and starch in the spike node were all decreased suggesting that the carbohydrates supporting the growth and development of the spike node were insufficient. Therefore, the number of large and small bundles, the area of large bundles, and sieve elements in spike nodes were decreased. As a result, the translocation ability of spike nodes was decreased. In addition, the activities of sucrose invertase and synthase in ears were also significantly decreased by W-B, which suggested that the capacity of ears to utilize sucrose was decreased. Thus the carbohydrate flux to ears was significantly impeded by W-B. Moreover, the competitive ability of apical florets was weaker than that of basal florets. Therefore, W-B inhibited the development of apical florets and reduced the growth rate of apical ovaries filaments more significantly than it impeded the growth rate of basal florets. As a result, the pollination time gap between basal and apical florets may be increased, leading to the abortion of apical kernels and the decrease in grain weight.

Significantly, the application of 6-BA effectively increased the content of cytokinin and decreased the JA content in the spike node and ears, thus increasing the activities of enzymes involved in the immediate sucrose metabolism. As a result, the structure of the spike node was improved, promoting the translocation of sucrose into the ears. Moreover, the ability of ears to unload and metabolize sucrose was increased. Therefore, the growth and development of spikelets and florets were more coordinated and synchronized. Consequently, the structure of filaments of apical florets was improved, the fertilization ability of apical florets was increased, and the fertilized florets, kernels per ear, and grain yield were increased.

## Conclusion

**FIGURE 9:**
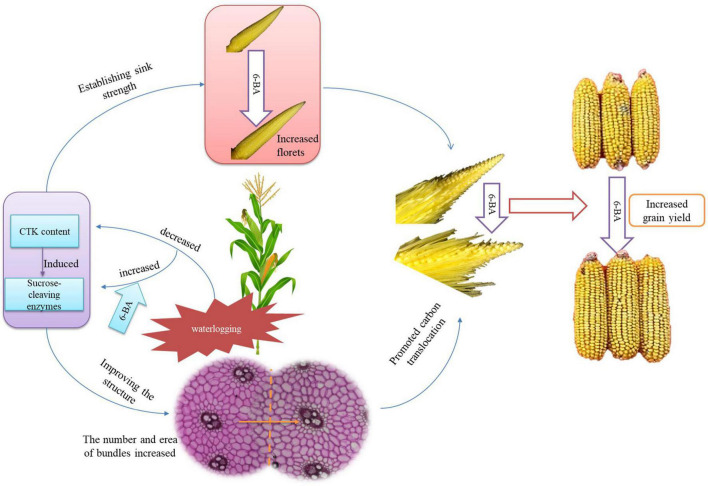
The mechanism of 6-BA improving the grain yield of summer maize. The application of 6-BA significantly improved the hormone and sugar metabolism, thus establishing the sink strength and improving the spike node structure to promote carbon translocation. As a result, the number of florets was increased; the spikelets and florets were developed coordinately and synchronously, leading to a higher grain yield of waterlogged summer maize.

The application of 6-BA increased the contents of cytokinin and SA and decreased the JA content during the crucial phase of floret differentiation, thus inducing the sucrose-cleaving enzymes and establishing the sink strength. Therefore, the number of florets was increased. Additionally, the application of 6-BA regulated the hormones during the phase of sexual organ formation, thus improving the carbon translocation ability of the spike node and increasing the utilization ability of the ears. Consequently, the spikelets and florets were developed coordinately and synchronously, thus improving the development of apical florets and the structure of filaments and increasing the fertilized florets, kernels per ear, and grain yield of summer maize (Figure 9).

## Data Availability Statement

The original contributions presented in the study are included in the article/Supplementary Material, further inquiries can be directed to the corresponding author/s.

## Author Contributions

JH: data curation, writing—original draft, visualization, and investigation. BR: writing—review and editing. YC, PL, and BZ: supervision. JZ: conceptualization, writing—review and editing, and funding acquisition. All authors contributed to the article and approved the submitted version.

## Conflict of Interest

The authors declare that the research was conducted in the absence of any commercial or financial relationships that could be construed as a potential conflict of interest.

## Publisher’s Note

All claims expressed in this article are solely those of the authors and do not necessarily represent those of their affiliated organizations, or those of the publisher, the editors and the reviewers. Any product that may be evaluated in this article, or claim that may be made by its manufacturer, is not guaranteed or endorsed by the publisher.

## Supplementary Material

Supplementary Figure 1 The phenotype of morphological differences in waterlogging maize treated with 6-BA and control (2017). CK, control, no waterlogging stress; CK-B, spraying 6-BA on non-waterlogged plants; W, waterlogging; W-B, spraying 6-BA after waterlogging.

Supplementary Figure 2 The effects of 6-BA on ear development of maize hybrid DH605 in 2018. CK, control, no waterlogging stress; CK-B, spraying 6-BA on non-waterlogged plants; W, waterlogging; W-B, spraying 6-BA after waterlogging.

Supplementary Figure 3 The difference of ears between 6-BA treatment and the control at the T1 and T2 stages. CK, control, no waterlogging stress; CK-B, spraying 6-BA on non-waterlogged plants; W, waterlogging; W-B, spraying 6-BA after waterlogging.

Supplementary Figure 4 The effects of 6-BA on the scan structure of filament of apical florets. CK, control, no waterlogging stress; CK-B, spraying 6-BA on non-waterlogged plants; W, waterlogging; W-B, spraying 6-BA after waterlogging.

Supplementary Figure 5 The structure of spike nodes. CK, control, no waterlogging stress; CK-B, spraying 6-BA on non-waterlogged plants; W, waterlogging; W-B, spraying 6-BA after waterlogging.

## References

[B1] Abid M. Tian Z. Hu J. Ullah A. Dai T. (2017). Activities of carbohydrate-metabolism enzymes in pre-drought primed wheat plants under drought stress during grain filling. *J. Integr. Plant Biol.* 46 783–795. 10.1111/jipb.12628 29266873

[B2] Akihiro T. Mizuno K. Fujimura T. (2005). Gene expression of ADP-glucose pyrophosphorylase and starch contents in rice cultured cells are cooperatively regulated by sucrose and ABA. *Plant Cell Physiol.* 46 937–946. 10.1093/pcp/pci101 15821022

[B3] Albacete A. Cantero-Navarro E. Balibrea M. E. Großkinsky D. K. de la Cruz González M. Martínez-Andújar C. (2014). Hormonal and metabolic regulation of tomato fruit sink activity and yield under salinity. *J. Exp. Bot.* 65 6081–6095. 10.1093/jxb/eru347 25170099 PMC4203140

[B4] Barbier F. Péron T. Lecerf M. Perez-Garcia M. D. Sakr S. (2015). Sucrose is an early modulator of the key hormonal mechanisms controlling bud outgrowth in rosa hybrida downloaded from. *J. Exp. Bot.* 66 2569–2582. 10.1093/jxb/erv047 25873679 PMC4986866

[B5] Bonnett O. T. (1940). Development of the staminate and pistillate inflorescence of sweet corn. *J. Agric. Res.* 60 25–37.

[B6] Brocard-Gifford I. Lynch T. J. Garcia M. E. Malhotra B. Finkelstein R. R. (2004). The *Arabidopsis thaliana* ABSCISIC ACID-INSENSITIVE 8 locus encodes a novel protein mediating abscisic acid and sugar responses essential for growth. *Plant Cell* 16 406–421. 10.1105/tpc.018077 14742875 PMC341913

[B7] Cárcova J. Otegui M. E. (2001). Ear temperature and pollination timing effects on maize kernel set. *Crop Sci.* 41 1809–1815. 10.2135/cropsci2001.1809 34798789

[B8] Chen W. S. Liu H. Y. Liu Z. H. Yang L. Chen W. H. (1994). Geibberllin and temperature influence carbohydrate content and flowering in Phalaenopsis. *Physiol. Plant.* 90 391–395. 10.1111/j.1399-3054.1994.tb00404.x

[B9] Choi J. Huh S. U. Kojima M. Sakakibara H. Paek K.-H. Hwang I. (2010). The cytokinin-activated transcription factor ARR2 promotes plant immunity via TGA3/NPR1-dependent salicylic acid signaling in *Arabidopsis*. *Dev. Cell* 19 284–295. 10.1016/j.devcel.2010.07.011 20708590

[B10] Choubane D. Rabot A. Mortreau E. Legourrierec J. Péron T. Foucher F. (2012). Photocontrol of bud burst involves gibberellin biosynthesis in *Rosa* sp. *J. Plant Physiol.* 169 1271–1280. 10.1016/j.jplph.2012.04.014 22749285

[B11] Cui H. Y. Camberato J. J. Jin L. B. Zhang J. W. (2015). Effects of shading on spike differentiation and grain yield formation of summer maize in the field. *Int. J. Biometeorol* 59 1189–1200. 10.1007/s00484-014-0930-5 25380975

[B12] Ehness R. Ecker M. Roitsch G. T. (1997). Glucose and stress independently regulate source and sink metabolism and defense mechanisms via signal transduction pathways involving protein phosphorylation. *Plant Cell Online* 9 1825–1841. 10.1105/tpc.9.10.1825 12237349 PMC157025

[B13] Engelberth J. Schmelz E. A. Alborn H. T. Cardoza Y. J. Huang J. Tumlinson J. H. (2003). Simultaneous quantification of jasmonic acid and salicylic acid in plants by vapor phase extraction and gas chromatography chemical ionization-mass spectrometry. *Anal. Biochem.* 312 242–250. 10.1016/s0003-2697(02)00466-9 12531212

[B14] Evert R. F. (1982). Sieve-tube structure in relation to function. *BioScience* 32 789–795. 10.2307/1308972

[B15] Finkelstein R. R. Gampala S. S. Rock C. D. (2002). Abscisic acid signaling in seeds and seedlings. *Plant Cell* 14 S15–S45. 10.1105/tpc.010441 12045268 PMC151246

[B16] Freier G. Vilella F. Hall A. J. (1984). Within-ear pollination synchrony and kernel set in maize. *Maydica* 29 317–324.

[B17] Gaille C. Kast P. Hass D. (2002). Salicylate biosynthesis in *Pseudomonas aeruginosa*. Purification and characterization of PchB, a novel bifunctional enzyme displaying isochorismate pyruvate-lyase and chorismate mutase activities. *J. Biol. Chem.* 277 21768–21775. 10.1074/jbc.M202410200 11937513

[B18] Gaille C. Reimmann C. Haas D. (2003). Isochorismate synthase (PchA), the first and rate-limiting enzyme in salicylate biosynthesis of *Pseudomonas aeruginosa*. *J. Biol. Chem.* 278 16893–16898. 10.1074/jbc.M212324200 12624097

[B19] Gao H. Wang P. Diao S. Cao L. (2007). Effect of 6-ba on dynamic changes of four endogenous hormones concentrations of prunus cerasus tissue culture seedlings. *J. Northeast For. Univ.* 35 46–48.

[B20] Gao X. Z. Wang Z. X. Xu J. F. (1989). *Relationship Between Yield and Grain Number Per Ear And 1000-Grain Weight Of Maize.* Jinan: Shandong Agricultural Sciences.

[B21] Hanft J. Jones M. R. J. (1986). Kernel abortion in maize: I. carbohydrate concentration patterns and acid invertase activity of maize kernels induced to abort in vitro. *Plant Physiol.* 81 503–510. 10.1104/pp.81.2.503 16664846 PMC1075366

[B22] Ho L. C. Lecharny A. Willenbrink J. (1991). “Sucrose cleavage in relation to import and metabolism of sugars in sink organs,” in *Recent Advances in Phloem Transport and Assimilate Compartmentation*, eds Bonnemain J. S. Delrot S. Lucas W. J. Dainty J. (Nantes: Ouest Editions), 178–186. 10.1626/pps.2.178

[B23] Hu J. Ren B. Dong S. Liu P. Zhao B. Zhang J. (2020). Comparative proteomic analysis reveals that exogenous 6-benzyladenine (6-BA) improves the defense system activity of waterlogged summer maize. *BMC Plant Biol.* 20:44. 10.1186/s12870-020-2261-5 31996151 PMC6988316

[B24] Hu J. Ren B. Dong S. Liu P. Zhao B. Zhang J. (2021a). Poor development of spike differentiation triggered by lower photosynthesis and carbon partitioning reduces summer maize yield after waterlogging. *Crop J.* 10.1016/j.cj.2021.08.001

[B25] Hu J. Ren B. Dong S. Liu P. Zhao B. Zhang J. (2021b). 6-Benzyladenine increasing subsequent waterlogging-induced waterlogging tolerance of summer maize by increasing hormone signal transduction. *Ann. N. Y. Acad. Sci.* 1509 89–112. 10.1111/nyas.14708 34766352

[B26] Jiang X. Li H. Wang T. Peng C. Wang H. Wu H. (2012). Gibberellin indirectly promotes chloroplast biogenesis as a means to maintain the chloroplast population of expanded cells. *Plant J.* 72 768–780. 10.1111/j.1365-313X.2012.05118.x 23020316

[B27] Julius B. T. Leach K. A. Tran T. M. Mertz R. A. Braun D. M. (2017). Sugar transporters in plants: new insights and discoveries. *Plant Cell Physiol.* 58 1442–1460. 10.1093/pcp/pcx090 28922744

[B28] Kang D. U. Zhao W. Q. Zhou Z. G. Shao J. J. Wei H. U. Kong L. J. (2021). Hormonal changes play important roles in the key period of superior and inferior earshoot differentiation in maize. *J. Agric. Sci.* 20:13.

[B29] Kiesselbach T. A. (1949). *The Structure and Reproduction of Corn. Research Bulletin 161.* Lincoln, NE: University of Nebraska.

[B30] Kuiper D. (1993). Sink strength: established and regulated by plant growth regulators. *Plant Cell Environ.* 16 1025–1026. 10.1111/j.1365-3040.1996.tb02052.x

[B31] Kushwah S. Laxmi A. (2014). The interaction between glucose and cytokinin signal transduction pathway in *Arabidopsis thaliana*. *Plant Cell Environ.* 37 235–253. 10.1111/pce.12149 23763631

[B32] Laby R. J. Kincaid M. S. Kim D. Gibson S. I. (2000). The *Arabidopsis* sugar-insensitive mutants sis4 and sis5 are defective in abscisic acid synthesis and response. *Plant J.* 23 587–596. 10.1046/j.1365-313x.2000.00833.x 10972885

[B33] LeClere S. Schmelz E. A. Chourey P. S. (2008). Cell wall invertase-deficient miniature1 kernels have altered phytohormone levels. *Phytochemistry* 69 692–699. 10.1016/j.phytochem.2007.09.011 17964617

[B34] Lesk C. Rowhani P. Ramankutty N. (2016). Influence of extreme weather disasters on global crop production. *Nature* 529:84. 10.1038/nature16467 26738594

[B35] Liu S. S. Chen J. Li S. C. Zeng X. Meng Z. X. Guo S. X. (2015). Comparative transcriptome analysis of genes involved in GA-GID1-DELLA regulatory module in symbiotic and asymbiotic seed germination of Anoectochilus roxburghii (Wall.) Lindl. (Orchidaceae). *Int. J. Mol. Sci.* 16 30190–30203. 10.3390/ijms161226224 26694378 PMC4691166

[B36] Machado R. A. Baldwin I. T. Erb M. (2017). Herbivory-induced jasmonates constrain plant sugar accumulation and growth by antagonizing gibberellin signaling and not by promoting secondary metabolite production. *New Phytol.* 215 803–812. 10.1111/nph.14597 28631319

[B37] Mehouachi J. Tadeo F. R. Zaragoza S. Primo-Millo E. Talon M. (1996). Effects of gibberellic acid and paclobutrazol on growth and carbohydrate accumulation in shoots and roots of citrus rootstock seedlings. *J. Hortic. Sci.* 71 747–754. 10.1080/14620316.1996.11515455

[B38] Miyamoto K. Ito E. Yamamoto H. Ueda J. Kamisaka S. (2000). Gibberellin-enhanced growth and sugar accumulation in growing subhooks of etiolated *Pisum sativum* seedlings: effects of actinomycin D on invertase activity, soluble sugars and stem elongation. *J. Plant Physiol.* 156 449–453. 10.1016/s0176-1617(00)80157-1

[B39] Miyamoto K. Ueda J. Kamisaka S. (1993). Gibberellin-enhanced sugar accumulation in growing subhooks of etiolated *Pisum sativum* seedlings. Effects of gibberellic acid, indoleacetic acid and cycloheximide on invertase activity, sugar accumulation and growth. *Physiol. Plant.* 88 301–306.

[B40] Moore C. E. Meacham-Hensold K. Lemonnier P. Slattery R. A. Cavanagh A. P. (2021). The effect of increasing temperature on crop photosynthesis: from enzymes to ecosystems. *J. Exp. Bot.* 72 2822–2844. 33619527 10.1093/jxb/erab090 PMC8023210

[B41] Parish R. W. Phan H. A. Sylvana I. Li S. F. (2012). Tapetal development and abiotic stress: a centre of vulnerability. *Funct. Plant Biol.* 39 553–559. 32480807 10.1071/FP12090

[B42] Ranwala A. P. Miller W. B. (2008). Gibberellin-mediated changes in carbohydrate metabolism during flower stalk elongation in tulips. *Plant Growth Regul.* 55 241–248.

[B43] Ren B. Z. Zhang J. W. Dong S. T. Liu P. Zhao B. (2017). Regulations of 6-benzyladenine (6-BA) on leaf ultrastructure and photosynthetic characteristics of waterlogged summer maize. *J. Plant Growth Regul.* 36 743–754.

[B44] Rohde A. Kurup S. Holdsworth M. (2000). ABI3 emerges from the seed. *Trends Plant Sci.* 5 418–419. 11203275 10.1016/s1360-1385(00)01736-2

[B45] Rook F. Corke F. Card R. Munz G. Smith C. Bevan M. W. (2001). Impaired sucrose-induction mutants reveal the modulation of sugar-induced starch biosynthetic gene expression by abscisic acid signalling. *Plant J.* 26 421–433. 11439129 10.1046/j.1365-313x.2001.2641043.x

[B46] Sakr S. Wang M. Dédaldéchamp F. Perez-Garcia M. D. Ogé L. Hamama L. (2018). The sugar-signaling hub: overview of regulators and interaction with the hormonal and metabolic network. *Int. J. Mol. Sci.* 19:2506. 10.3390/ijms19092506 PMC6165531 30149541

[B47] Schussler J. R. Westgate M. E. (1994). Increasing assimilate reserves does not prevent kernel abortion at low water potential in maize. *Crop Sci.* 34 1074–1080.

[B48] Setter T. L. Waters I. (2003). Review of prospects for germplasm improvement for waterlogging tolerance in wheat, barley and oats. *Plant Soil* 253 1–34.

[B49] Shen S. Zhang L. Liang X. G. Zhao X. Lin S. Qu L. H. (2018). Delayed pollination and low availability of assimilates are major factors causing maize kernel abortion. *J. Exp. Bot.* 69 1599–1613. 29365129 10.1093/jxb/ery013 PMC5888920

[B50] Takei K. Yamaya T. Sakakibara H. (2004). *Arabidopsis* CYP735A1 and CYP735A2 encode cytokinin hydroxylases that catalyze the biosynthesis of trans-zeatin. *J. Biol. Chem.* 279 41866–41872. 15280363 10.1074/jbc.M406337200

[B51] Tao F. Yokozawa M. Hayashi Y. Lin E. (2003a). Changes in soil moisture in China over the last half-century and their effects on agricultural production. *Agric. For. Meteorol.* 118 251–261.

[B52] Tao F. Yokozawa M. Hayashi Y. Lin E. (2003b). Future climate change, the agricultural water cycle, and agricultural production in China. *Agric. Ecosyst. Environ.* 95 203–215.

[B53] Tao F. Yokozawa M. Liu J. Zhang Z. (2008). Climate-crop yield relationships at province scale in China and the impacts of recent climate trend. *Clim. Res.* 38 83–94.

[B54] Thompson B. (2014). Genetic and hormonal regulation of maize inflorescence development. *Adv. Bot. Res.* 72 263–296.

[B55] Tsai Y. C. Weir N. R. Hill K. Zhang W. Kim H. J. Shiu S.-H. (2012). Characterization of genes involved in cytokinin signaling and metabolism from rice. *Plant Physiol.* 158 1666–1684. 22383541 10.1104/pp.111.192765 PMC3320177

[B56] Tuna A. L. Kaya C. Dikilitas M. Higgs D. (2008). The combined effects of gibberellic acid and salinity on some antioxidant enzyme activities, plant growth parameters and nutritional status in maize plants. *Environ. Exp. Bot.* 62 1–9.

[B57] Vishwakarma K. Upadhyay N. Kumar N. Yadav G. Singh J. Mishra R. K. (2017). Abscisic acid signaling and abiotic stress tolerance in plants: a review on current knowledge and future prospects. *Front. Plant Sci.* 8:161. 28265276 10.3389/fpls.2017.00161 PMC5316533

[B58] Wang G. Zhang G. Wu M. (2016). CLE peptide signaling and crosstalk with phytohormones and environmental stimuli. *Front. Plant Sci.* 6:1211. 26779239 10.3389/fpls.2015.01211 PMC4703810

[B59] Wang Y. (2019). *Inverse Development of Maize Inflorescence andin Situ Analysis of Hormones.* Shenyang: Shenyang Agricultural Universty.

[B60] Wildermuth M. C. Dewdney J. Wu G. Ausubel F. M. (2001). Isochorismate synthesis is required to synthesize salicylic acid for plant defense. *Nature* 414 562–565. 11734859 10.1038/35107108

[B61] Xu J. Henry A. Sreenivasulu N. (2020). Rice yield formation under high day and night temperatures—a prerequisite to ensure future food security. *Plant Cell Environ.* 43 1595–1608. 32112422 10.1111/pce.13748

[B62] Zheng Z. L. Hu Y. H. Li B. H. (1990). Studies on interrelationships between the type, shape, and critical stage of female ear florets in maize (*zea mays* L.) and kernal formation. *J. Agric. Univ. Hebei* 13 34–38.

