# Supplementary figures and images for: Exogenous 6-Benzyladenine Improved the Ear Differentiation of Waterlogged Summer Maize by Regulating the Metabolism of Hormone and Sugar
Source: Front Plant Sci. 2022 Apr 7;13:848989. doi: 10.3389/fpls.2022.848989 (PMC9021890; doi:10.3389/fpls.2022.848989)

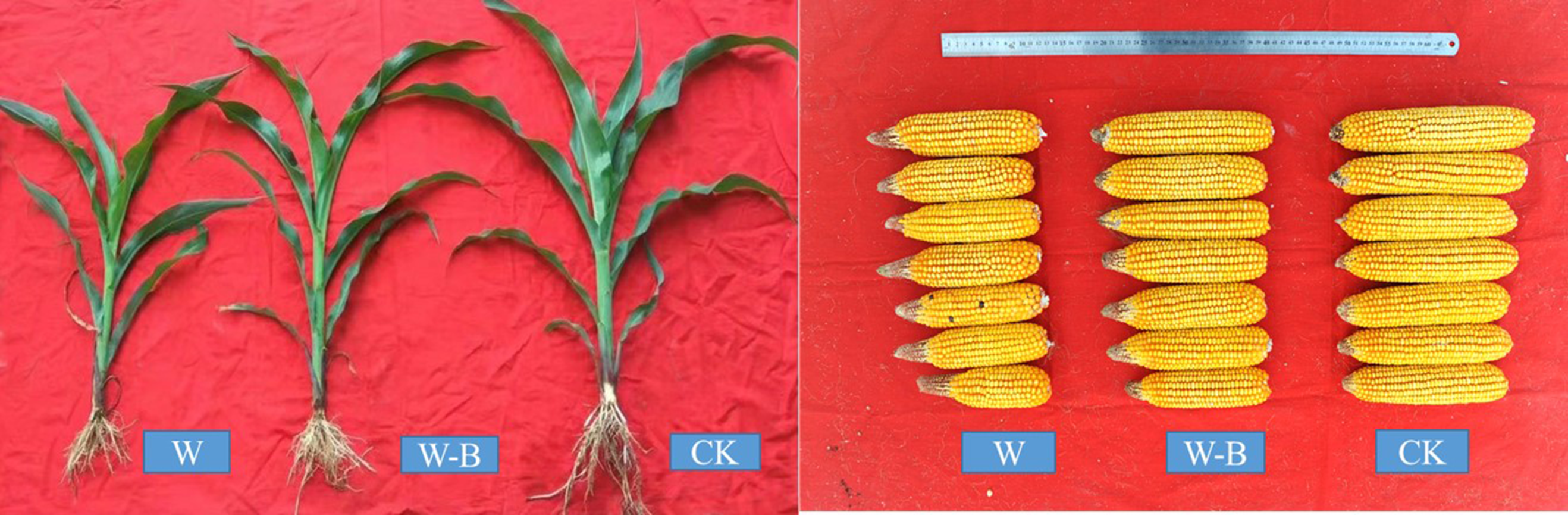

Supplement: Supplementary Figure 1 — The phenotype of morphological differences in waterlogging maize treated with 6-BA and control (2017). CK, control, no waterlogging stress; CK-B, spraying 6-BA on non-waterlogged plants; W, waterlogging; W-B, spraying 6-BA after waterlogging. [file Image_1.tif]

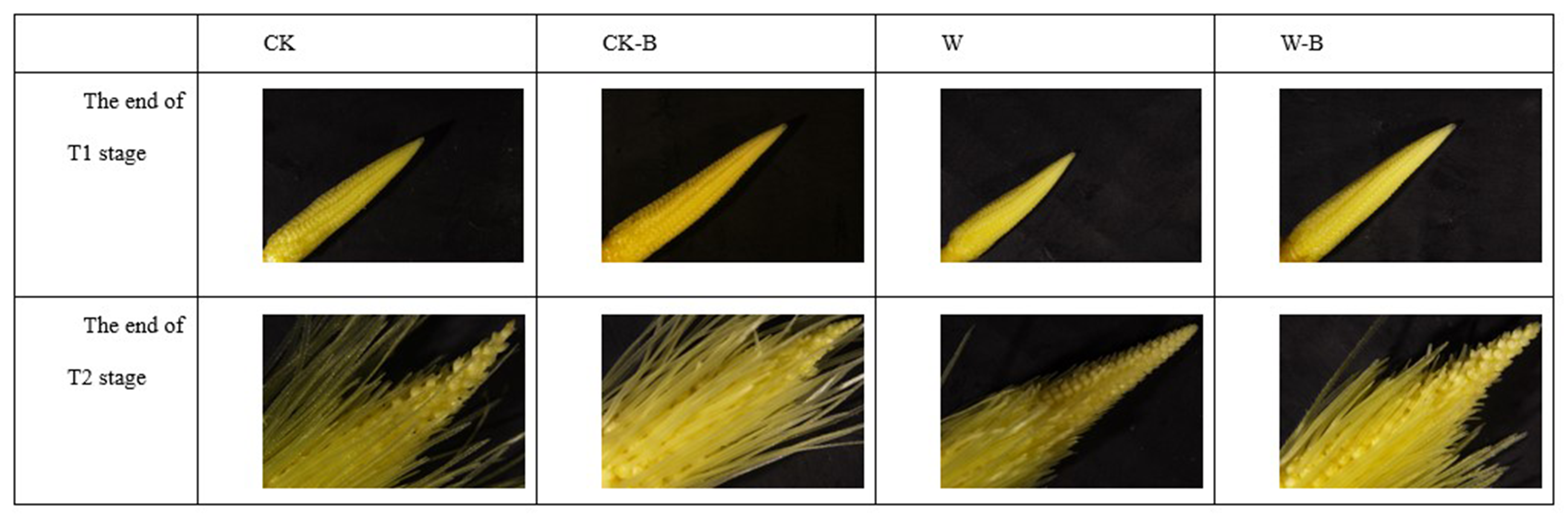

Supplement: Supplementary Figure 2 — The effects of 6-BA on ear development of maize hybrid DH605 in 2018. CK, control, no waterlogging stress; CK-B, spraying 6-BA on non-waterlogged plants; W, waterlogging; W-B, spraying 6-BA after waterlogging. [file Image_2.tif]

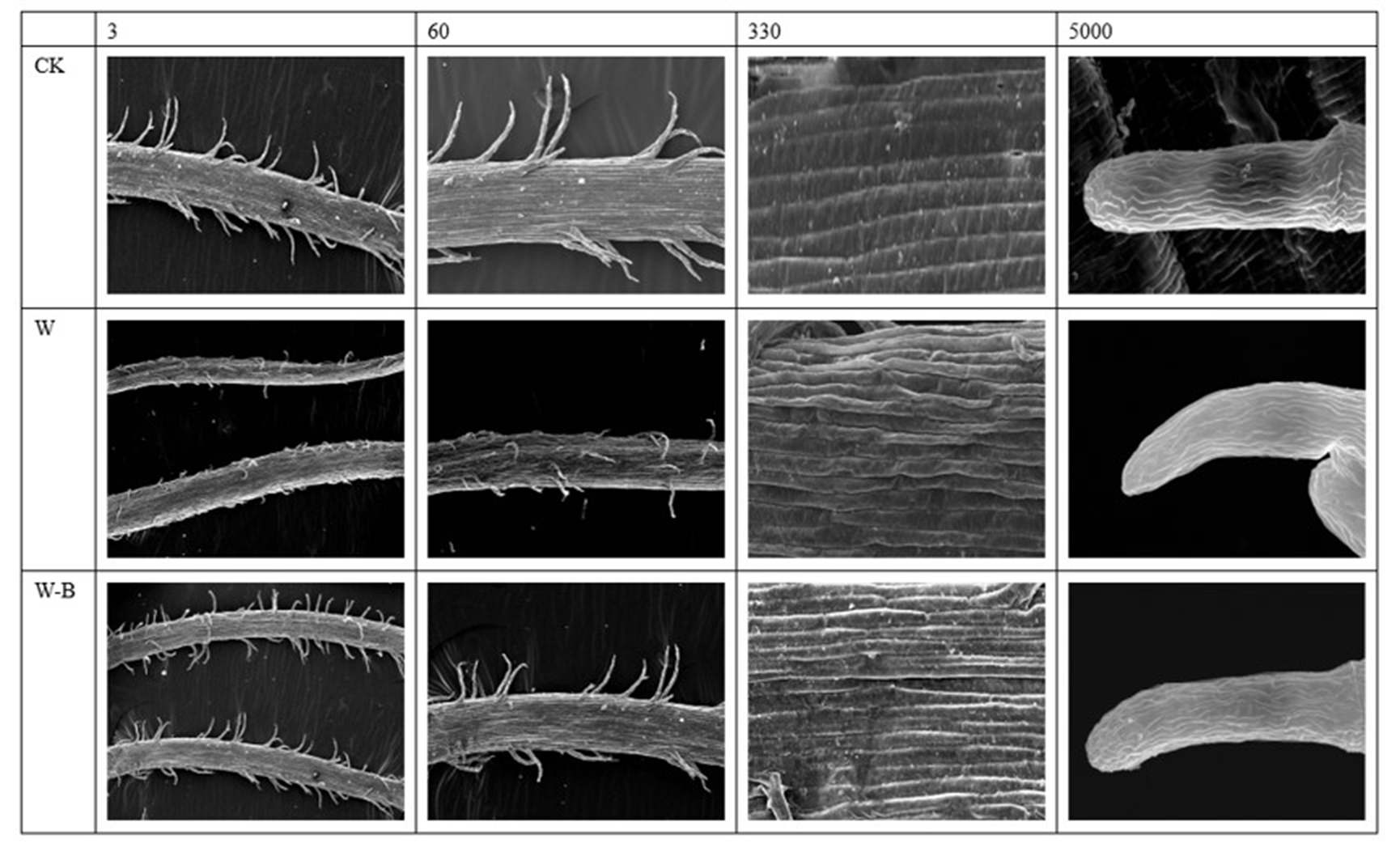

Supplement: Supplementary Figure 4 — The effects of 6-BA on the scan structure of filament of apical florets. CK, control, no waterlogging stress; CK-B, spraying 6-BA on non-waterlogged plants; W, waterlogging; W-B, spraying 6-BA after waterlogging. [file Image_4.tif]

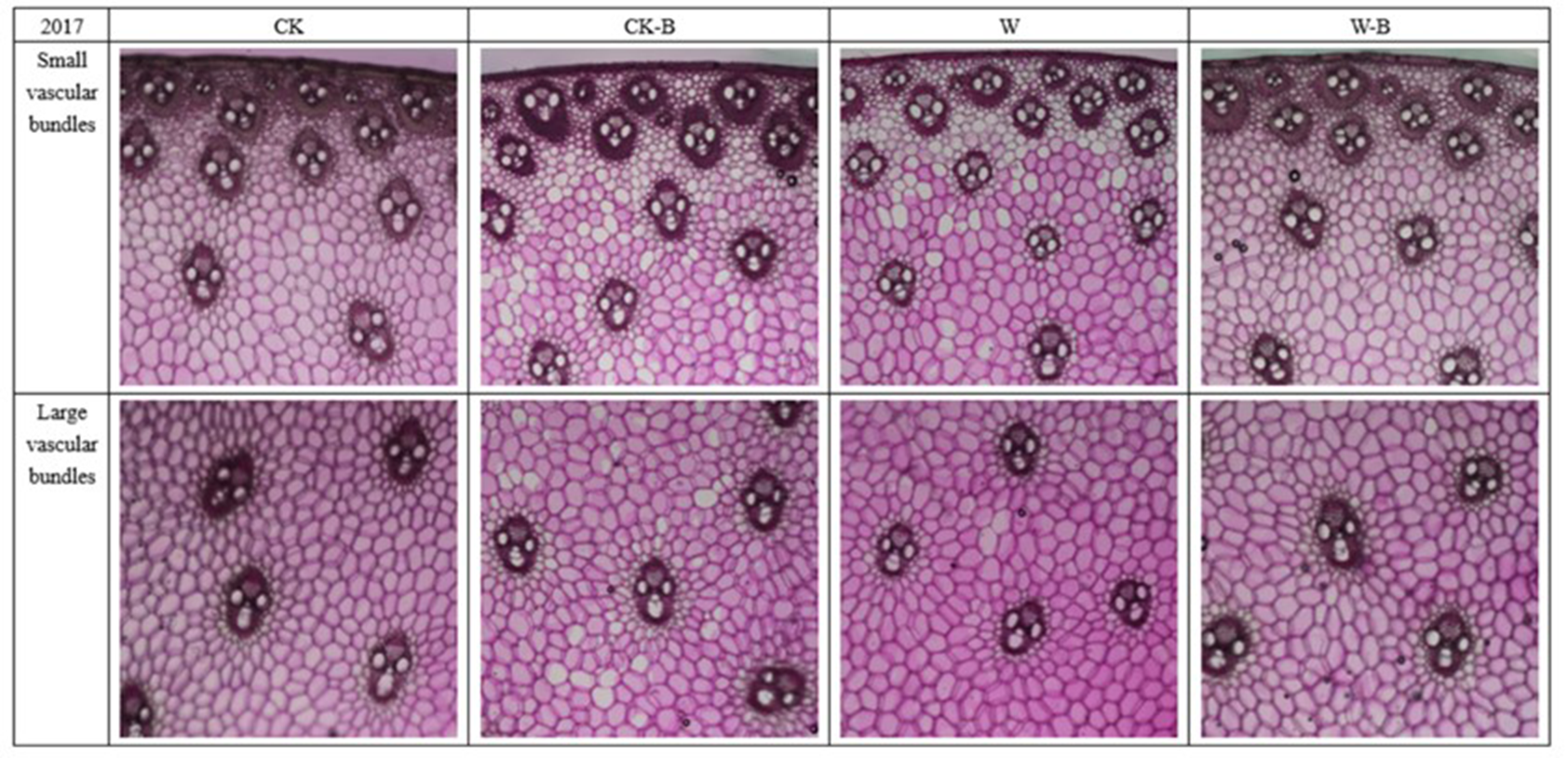

Supplement: Supplementary Figure 5 — The structure of spike nodes. CK, control, no waterlogging stress; CK-B, spraying 6-BA on non-waterlogged plants; W, waterlogging; W-B, spraying 6-BA after waterlogging. [file Image_5.tif]
